# Natural Monoterpenes as Potential Therapeutic Agents against Atherosclerosis

**DOI:** 10.3390/ijms24032429

**Published:** 2023-01-26

**Authors:** Jing Yang, Chao Zhong, Jun Yu

**Affiliations:** 1 Center for Translational Medicine, College of Traditional Chinese Medicine, Jiangxi University of Chinese Medicine, Nanchang 330004, China; 2 Department of Cardiovascular Sciences, Center for Metabolic Disease Research, Lewis Katz School of Medicine, Temple University, Philadelphia, PA 19140, USA

**Keywords:** natural products, monoterpenes, atherosclerosis, atheroprotective effect, antiatherogenic drugs

## Abstract

Traditional herbal medicines based on natural products play a pivotal role in preventing and managing atherosclerotic diseases, which are among the leading causes of death globally. Monoterpenes are a large class of naturally occurring compounds commonly found in many aromatic and medicinal plants. Emerging evidence has shown that monoterpenes have many biological properties, including cardioprotective effects. Remarkably, an increasing number of studies have demonstrated the therapeutic potential of natural monoterpenes to protect against the pathogenesis of atherosclerosis. These findings shed light on developing novel effective antiatherogenic drugs from these compounds. Herein, we provide an overview of natural monoterpenes’ effects on atherogenesis and the underlying mechanisms. Monoterpenes have pleiotropic and multitargeted pharmacological properties by interacting with various cell types and intracellular molecular pathways involved in atherogenesis. These properties confer remarkable advantages in managing atherosclerosis, which has been recognized as a multifaceted vascular disease. We also discuss limitations in the potential clinical application of monoterpenes as therapeutic agents against atherosclerosis. We propose perspectives to give new insights into future preclinical research and clinical practice regarding natural monoterpenes.

## 1. Introduction

Atherosclerosis is the major underlying pathological basis for coronary artery disease, cerebrovascular disease, and peripheral arterial disease, which causes significant morbidity and mortality worldwide [1,2,3,4]. A spectrum of mechanisms is involved in the initiation and progression of atherosclerosis, including endothelial dysfunction, abnormal lipid metabolism, oxidative stress, and inflammation [3,5,6]. Additionally, a wide array of risk factors such as dyslipidemia, obesity, diabetes, hypertension, aging, and smoking are recognized to be associated with atherosclerosis [7], and the interplay between them makes atherosclerosis a complex condition. Over the past decades, despite significant advances in prevention therapies using contemporary intervention and pharmacologic agents against atherosclerosis, the burden of ischemic cardiovascular conditions remains substantial [8,9]. Therefore, to better cope with the life-threatening atherosclerotic cardiovascular disease (ASCVD), it is urgent to seek new effective therapeutic agents targeting atherosclerosis without marked side effects.

Natural products derived from plants remain attractive sources of molecular entities for developing novel medicinal and therapeutic agents [10]. Due to fewer adverse effects and lower cost of natural compounds compared with chemotherapeutic agents, therapies based on natural products have long been widely used in traditional medicines to treat ASCVD in China and many other Asian countries [11,12]. Terpenes represent a large class of plant-derived secondary metabolites with five-carbon isoprene (C5H8) units as their primary structural component [13]. According to the number of isoprene units within their chemical structure, terpenes can be classified into hemiterpene (C5), monoterpene (C10), sesquiterpene (C15), diterpene (C20), sesterpene (C25), triterpene (C30), and polyterpene (>C30) [13]. The monoterpenes, a major chemical group of terpenes with two isoprene units in their structure, are commonly found in many bioactive essential oils and medicinal plants [14]. It has been well documented that monoterpenes have many biological properties, including anti-bacterial, anti-fungal, anti-oxidative, anti-inflammatory, and anti-tumor activities [14]. Moreover, they are widely utilized in the pharmaceutical preparations, food, and cosmetic industries [15]. Notably, a growing body of evidence has shown that monoterpenes are promising in their potential roles in protecting against cardiovascular disease. For example, geniposide, a well-known iridoid glycoside, has been reported to have remarkable therapeutic potential in managing cardiac fibrosis, cardiac hypertrophy, myocardial ischemia/reperfusion injury, obesity-related cardiac injury, atherosclerosis, ischemic stroke, and hypertension. This makes it an attractive candidate for cardiovascular medicine [16]. For a detailed overview of the protective roles of monoterpenoids in the cardiovascular system, we refer to an excellent recent review [17]. In light of the therapeutic potential of monoterpenes in cardiovascular disease, they are involved in an increasing number of patents registered within the cardiovascular field, highlighting the significant role of these natural compounds in developing new drugs intended to prevent and manage cardiovascular disease [18].

**Figure 1:**
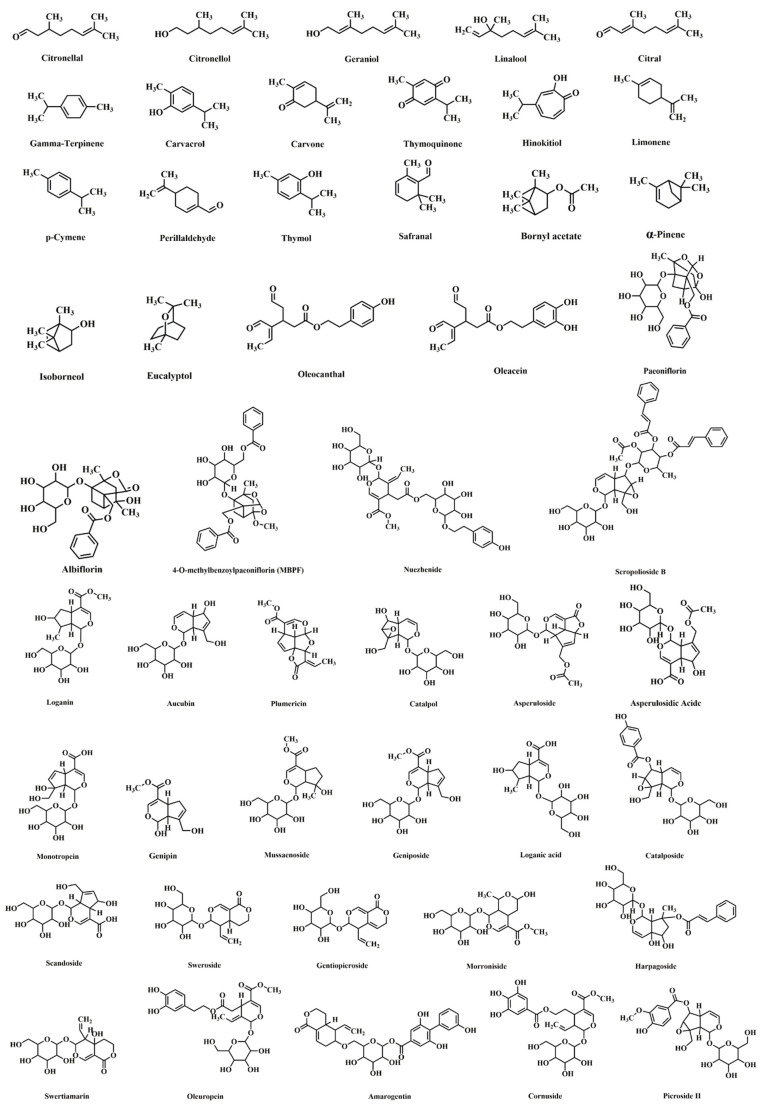
Chemical structures of natural monoterpenes with potential antiatherogenic effects.

As mentioned above, atherosclerosis is the major contributor to CVD. Targeting atherosclerosis is of fundamental importance for counteracting ASCVD. In recent years, investigations on medicinal natural products have led to increased attention to the potential of naturally occurring monoterpenes and their derivatives in treating atherosclerosis. Various cellular and molecular targets of monoterpenes have also been identified. These findings shed light on the mechanisms by which monoterpenes protect against atherosclerosis in the clinic. However, there needs to be detailed reviews on the research progress of monoterpenes in the context of atherosclerosis. In this review, we overview the pharmacological effects of natural monoterpenes on atherosclerosis, focusing on the underlying cellular and molecular mechanisms. A total of 48 natural monoterpenes were included in this review, and their chemical structures are shown in Figure 1. We attempt to provide insights into the development of monoterpene-based pharmacotherapies to prevent and treat atherosclerosis and its related clinical complications.

## 2. Natural Monoterpenes Modulate Serum Lipid Profile 

Hyperlipidemia refers to the dysregulated lipid metabolism manifesting high levels of total cholesterol (TC), triglycerides (TG), and low-density lipoprotein cholesterol (LDL-C) and a decreased level of high-density lipoprotein cholesterol (HDL-C) in the circulation. Hyperlipidemia is considered a prominent risk factor for the pathophysiology of atherosclerosis [19]. In the early stage of atherosclerotic lesions, LDL particles accumulate and undergo modification in the intima of the arterial wall, leading to subsequent monocyte recruitment and cholesterol-laden foam cell formation [3]. Multiple lines of evidence from experimental and clinical studies have established that excessive serum LDL-C is not merely associated with high risk but also a direct underlying mechanism of atherosclerosis [20]. Thus, LDL-C is currently an essential target for the intervention of ASCVD. The introduction of statin drugs, which effectively reduce LDL-C, has been the cornerstone for managing hyperlipidemia and ASCVD risk [9]. Nonetheless, safety issues related to statin therapy remain a chief concern because of the associated adverse effects, such as myopathy and hepatotoxicity [21], illustrating the need for new therapeutic strategies. 

3-hydroxy-3-methylglutaryl-coenzyme A (HMG-CoA) reductase, the rate-limiting enzyme in the biosynthesis of cholesterol, has been a promising target for developing hypolipidemic drugs such as the statins [3]. The expression of HMG-CoA reductase is critically regulated by sterol regulatory element binding protein-2 (SREBP-2), a crucial transcription factor controlling cellular cholesterol homeostasis [22]. HMG-CoA reductase is also post-transcriptionally regulated by the ubiquitin-proteasome system, which depends on the insulin-induced gene 1 (Insig) protein and the ubiquitin ligase gp78 [23]. A growing body of evidence has demonstrated that many natural monoterpenes improve hypercholesterolemia by targeting HMG-CoA reductase. Linalool is a naturally occurring monoterpene present in essential oils of various aromatic medicinal plants. Oral administration of linalool significantly alleviated high-fat diet (HFD)-induced hyperlipidemia in mice by diminishing plasma TC, TG, and LDL-C with a concomitant reduction of HMG-CoA reductase expression [24]. Furthermore, mechanistic studies have found that linalool could reduce the expression of SREBP-2 and enhance Insig expression and ubiquitination of HMG-CoA reductase, thus attenuating SREBP-2-mediated HMG-CoA reductase transcription and accelerating ubiquitin-dependent proteolysis of HMG-CoA reductase [24]. Moreover, as a critical energy sensor of cell metabolism, the AMP-activated protein kinase (AMPK) has been implicated in suppressing HMG-CoA reductase. It thus has therapeutic importance for treating hypercholesterolemia [25]. Administration of monoterpenes, such as amarogentin, oleuropein, and aucubin, significantly reduces serum TC and LDL-C by activating AMPK, suggesting that the modulation of AMPK/HMG-CoA reductase signaling may contribute to the hypocholesterolemic property of these monoterpenes [26,27,28]. 

Reverse cholesterol transport (RCT) refers to the delivery of accumulated cholesterol from the blood and the peripheral tissue into the liver for excretion. Manipulation of this process is thus expected to achieve the hypocholesterolemic effect [29]. It has been known that low-density lipoprotein receptor (LDLR), scavenger receptor class B type 1 (SR-B1), and ATP-binding cassette G1 (ABCG1) are involved in RCT. Thymoquinone, the major bioactive component in *Nigella sativa* volatile oil, could serve as a cholesterol-lowering agent by increasing the uptake of serum LDL-C via elevation of hepatic LDLR expression and by inhibiting HMG-CoA reductase-mediated cholesterol synthesis [30]. Geniposide, a well-known monoterpenoid derived from the fruit of *Gardenia jasminoides*, was found to attenuate cholesterol accumulation in the plasma and the liver, at least partly through facilitating RCT via upregulation of LDLR, SR-B1, and ABCG1 in the liver [31]. Another critical aspect of cholesterol metabolism is converting cholesterol into bile acids and their subsequent excretion. These processes are critically orchestrated by the farnesoid X receptor (FXR). Specifically, when abundant bile acids are produced, FXR-mediated negative feedback regulation suppresses hepatic bile acid synthesis while accelerating ileal bile acid reabsorption [32,33]. Therefore, by inducing bile acid synthesis and inhibiting bile acid reabsorption through FXR suppression, more cholesterol can be converted into bile acids with a concomitant enhancement of bile acids excretion, eventually leading to decreased cholesterol and thus improving atherosclerosis. Notably, a recent study showed that FXR suppression is an important mechanism underlying the protective effects of geniposide on cholesterol homeostasis and atherosclerosis, in addition to the regulation of RCT, as mentioned above [31]. The administration of geniposide significantly modulated the FXR-small heterodimer partner (SHP)-hepatocyte nuclear factor 4 (HNF-4α)/liver receptor homolog-1 (LRH-1) axis in the liver and the FXR/ileal bile acid-binding protein (I-BABP) axis in the ileum. These effects were associated with the induction of bile acid synthesis and excretion processes in animals fed with or without HFD, suggesting that geniposide exerts a hypocholesterolemic effect by regulating FXR-mediated liver-gut crosstalk of bile acids [31]. Similarly, elevation of bile acid excretion and improved serum lipid profile were observed in a rat model of hypercholesterolemia after oral administration of swertiamarin, which is the main constituent of plants such as *Enicostemma littorale*, making swertiamarin a potent lipid-lowering and atheroprotective agent [34]. 

Other monoterpenes [35,36,37,38,39,40,41,42,43,44,45,46,47] that modify serum lipid profiles are listed in Table 1. However, the detailed mechanism of their hypocholesterolemic property remains elusive for many. 

## 3. Natural Monoterpenes Protect against Atherosclerosis by Targeting Endothelial Cells

The endothelial cells are integral to the cardiovascular system and function as gatekeepers of vascular health and homeostasis. The dysfunction of vascular endothelial cells has been recognized as a critical component in the pathophysiology of atherosclerosis [48]. Mediated by endothelial dysfunction, circulating lipoprotein particles enter the artery wall, facilitating the recruitment of monocytes/macrophages and the formation of atherogenic foam cells, ultimately triggering a series of complex pathogenic processes to promote plaque formation [48]. 

### 3.1. Attenuation of Endothelial Pro-Inflammatory Activation

Triggered by various cardiovascular risk factors, endothelial cells undergo morphological and functional modifications, termed endothelial activation, manifesting increased expression of adhesion molecules such as vascular cell adhesion molecule-1 (VCAM-1), intercellular adhesion molecule-1 (ICAM-1), E-Selectin, and chemokines/pro-inflammatory cytokines such as interleukin-8 (IL-8), IL-6, tumor necrosis factor-α (TNF-α), and IL-1β. The results of endothelial activation are increased leukocyte adhesion and infiltration into the vascular wall leading to the propagation and development of vascular inflammation [48].

Nuclear factor κB (NF-κB) is a master transcription factor responsible for inflammatory responses [49]. Previous studies have demonstrated that NF-κB inactivation is an essential mechanism of the anti-endothelial activation effect of natural monoterpenes (Table 2). Peroxisome proliferator-activated receptor Gama (PPARγ) can attenuate inflammatory responses in the cardiovascular system, including endothelial cells. Growing evidence suggests that PPARγ is an upstream regulator of NF-κB in the anti-inflammatory process [50,51]. Eucalyptol is a monoterpene found naturally in many aromatic plants with anti-inflammatory effects. Pre-treatment with eucalyptol has been reported to suppress the expression of VCAM-1, E-selectin, IL-8, and IL-6 in lipopolysaccharide (LPS)-induced human umbilical vein endothelial cells (HUVECs), and this was achieved by blockade of NF-κB signaling. Importantly, by using PPARγ inhibitor or PPARγ gene silencing, LPS-induced activation of NF-κB and expression of inflammatory mediators in HUVECs could be reversed, suggesting that modulation of the PPARγ/NF-κB axis contributes to eucalyptol-mediated suppression of endothelial pro-inflammatory activation [52]. Similarly, several studies have shown that monoterpenes, including citral, citronellol, and genipin, can inhibit adhesion molecule expression in HUVECs and neutrophil/monocyte–endothelial cell adhesion by regulating the PPARγ-dependent NF-κB signaling pathway [53,54,55]. High mobility group box 1 (HMGB1) is a damage-associated molecular pattern (DAMP), secreted from endothelial cells and leukocytes that mediate inflammation, and correlates with the severity of atherosclerosis [56]. Paeoniflorin and cornuside, two natural monoterpenoids used in traditional oriental herbal medicine, were found to suppress the expression and release of HMGB1 in LPS- or lysophosphatidylcholine (LPC)-stimulated HUVECs, paralleling with reduced expression of endothelial cell-derived adhesion molecules and inflammatory factors [57,58]. The underlying mechanism may involve the induction of sirtuin 1 (SIRT1), a nicotinamide adenine dinucleotide–dependent protein deacetylase, which plays an important role in deacetylation of HMGB1 and thus inhibits HMGB1 release and subsequent NF-κB activation in HUVECs [57]. Additionally, reactive oxygen species (ROS) have been reported to induce the activation of NF-κB to promote the adhesiveness of endothelial cells [59]. Using a high glucose-induced HUVECs model, Wang et al. showed that geniposide exhibited a beneficial role in normalizing endothelial pro-inflammatory activation by inhibiting ROS overproduction, NF-κB activation, and monocyte–endothelial cell adhesion [60]. Recently, molecular docking analysis combined with in vitro cell culture-based approaches showed that amarogentin isolated from *Gentianaceace* plants could directly interact with AMPK to block NF-κB-mediated endothelial inflammation, indicating the pivotal role of AMPK/NF-κB in the protective effect of amarogentin on endothelial activation [26]. Notably, many monoterpenes with NF-κB inhibitory effects, such as cornuside, paeoniflorin, and geniposide, can attenuate endothelial activation through mitogen-activated protein kinase (MAPK) signaling (p38 MAPK, c-Jun N-terminal kinase (JNK), and extracellular signal-regulated kinase (ERK)), another major signaling pathway driving inflammatory response [57,61,62]. 

Endoplasmic reticulum (ER) stress, autophagy, and inflammation are tightly integrated biological processes in atherosclerosis’s pathogenesis [79]. ER homeostasis is crucial in determining cell survival or death based on the cellular stress factors present. When stimulated by pathological insults, intracellular unfolded protein responses can be induced to protect ER homeostasis. However, excessive and prolonged stimuli disturbing ER homeostasis can cause persistent ER stress responses, triggering inflammatory cascades and cell death events [79]. Thus, ER stress is acknowledged as a danger signal for inflammation. Paeoniflorin extracted from the traditional Chinese herb *Paeonia lactiflora* was reported to be able to modulate ER stress during endothelial pro-inflammatory activation [65]. Pre-treatment with paeoniflorin significantly reduced the expression of ER stress markers glucose regulated protein 78 (GRP78), C/EBP homologous protein (CHOP), and spliced X-box binding protein-1 (XBP-1), as well as improved ultrastructural abnormalities of the ER. These events ultimately contributed to the attenuation of NF-κB-dependent production of inflammatory mediators in LPS-induced HUVECs [65]. Similarly, catalpol, another natural monoterpene, could also alleviate endothelial inflammation, at least in part, by inhibiting ER stress [66]. Autophagy is an evolutionarily conserved lysosomal catabolic process engaged in degrading dysfunctional or surplus protein aggregates and organelles to maintain cellular homeostasis. Nevertheless, aberrant autophagy causes detrimental effects on cellular homeostasis and thus leads to the induction of inflammation and facilitates the pathophysiology of atherosclerosis [79]. In addition to regulating ER stress, paeoniflorin was found to promote autophagy and attenuate endothelial pro-inflammatory activation through a SIRT1-dependent mechanism [64]. 

Other monoterpenes [63,67,68,69,70,71,72,73,74,75,76,77,78] that attenuate endothelial pro-inflammatory activation are shown in Table 2. 

### 3.2. Inhibition of Endothelial Oxidative Stress 

Oxidative stress refers to an imbalance favoring the production of ROS over intrinsic antioxidant mechanisms, which leads to extensive cellular and molecular damage. Endothelial cells are susceptible to risk factors of oxidative stress, such as oxidized lipids, homocysteine, angiotensin II, hyperglycemia, and inflammatory mediators. These risk factors result in the overproduction of ROS followed by endothelial cell damage, dysfunction, pro-inflammatory activation, and apoptosis, eventually promoting the development of atherosclerosis [80]. Accordingly, targeting endothelial oxidative stress by attenuating ROS overproduction and/or improving intracellular antioxidant activity would be beneficial for protecting against endothelial oxidative injury and atherosclerosis. 

Studies have demonstrated that many monoterpenes can alleviate endothelial oxidative stress by either inhibiting pro-oxidant enzymes or by enhancing antioxidant enzymes to maintain cellular redox balance. For example, geraniol, paeoniflorin and harpagoside could suppress endothelial ROS production through the downregulation of NADPH oxidases (NOXs) and cyclooxygenase (COX), which are the predominant sources of ROS in the vasculature [81,82,83]. Other monoterpenes, such as thymoquinone, perillaldehyde, citronellal, geniposide, and monotropein, mitigate endothelial oxidative stress by strengthening the activities of antioxidant enzymes, including superoxide dismutase (SOD), glutathione peroxidase (GSH-Px), and catalase (CAT) [67,77,84,85,86]. Many monoterpenes exert anti-oxidative stress effects via attenuating pro-oxidant pathways and inducing antioxidant mechanisms (Table 3). 

The nuclear factor E2-related factor 2 (Nrf2) is a central transcription factor responsible for intracellular redox homeostasis [80]. Under basal conditions, Nrf2 binds to Kelch ECH associating protein 1 (Keap1) in the cytoplasm, promoting Nrf2 ubiquitination and degradation. Upon oxidative stress stimulation, Nrf2 dissociates from Keap1 and translocates into the nucleus, thereby inducing the expressions of phase II detoxifying enzymes and antioxidant enzymes for cellular defense [80]. A recent study demonstrated that paeoniflorin, a traditional Chinese herbal substance belonging to monoterpenoid, suppressed mitochondrial ROS production and restored mitochondrial functional damage in tert-butyl hydroperoxide (TBHP)-induced HUVECs [90]. Furthermore, a mechanistic study indicated that paeoniflorin directly interacted with cytoplasm Nrf2, resulting in the nuclear translocation of Nrf2 and activation of Nrf2-mediated antioxidant signaling, thus relieving TBHP-induced endothelial oxidative stress [90]. Another traditional Chinese herbal component with atheroprotective effects, geniposide, was found to attenuate H_2_O_2_-induced endothelial oxidative stress by activating the Nrf2 antioxidant pathway. Different from the paeoniflorin mentioned above, geniposide stimulated Nrf2 nuclear translocation by modulating AMPK/mechanistic target of rapamycin (mTOR) signaling [68]. Similarly, monoterpenes, such as eucalyptol, geraniol, and catalpol, have also been shown to have anti-oxidative stress effects via Nrf2 activation [42,87,92]. miR-21 is an endogenous miRNA implicated in various pathophysiological processes, including cardiovascular disorders [99]. Accumulating evidence suggests that the miR-21/phosphatase and tensin homolog (PTEN) pathway regulates cell survival and death in the cardiovascular system, including endothelial cells, which makes miR-21/PTEN a therapeutic target for cardiovascular disease [100,101]. Zhou et al. reported that pre-treatment with geniposide decreased ox-LDL-induced oxidative stress in HUVECs by reducing NOX2 expression and by upregulating antioxidant enzyme activities [67]. Using gain- and loss-of-function approaches, the investigators further revealed that geniposide could modulate the miR-21/PTEN pathway to restore the balance between intracellular oxidant and antioxidant states, thus preventing ox-LDL-induced endothelial oxidative injury [67]. Lectin-like oxidized low-density lipoprotein receptor-1 (LOX-1) is a well-known receptor for ox-LDL that plays a vital role in atherosclerosis. Homocysteine, a risk factor of atherosclerosis, has been reported to induce the expression of LOX-1 in endothelial cells and to promote ROS generation and oxidative injury [102,103]. Picroside II, a monoterpenoid isolated from traditional Chinese medicine *Picrorhiza scrophulariiflora*, was reported to counteract homocysteine-induced LOX-1 expression in a SIRT1-dependent manner and thus ameliorated HUVEC oxidative stress [76]. In addition, catalpol and monotropein, two naturally occurring monoterpenoids, were found to attenuate endothelial oxidative stress, at least in part, by inhibiting NF-κB activation, suggesting inflammation as a target of these compounds to regulate oxidative stress [66,77].

Other monoterpenes [89,91,95,97,98] that inhibit endothelial oxidative stress are listed in Table 3.

### 3.3. Modulation of Nitric Oxide (NO) Pathway

Endothelium-derived NO plays essential roles in normal endothelial functions. NO is a multifunctional signaling molecule controlling cardiovascular homeostasis, including regulating vasomotor tone, modulation of platelet activation and leukocyte adhesion, and manipulating local cell growth [104]. All established risk factors for atherosclerosis, such as hyperlipidemia, diabetes mellitus, hypertension, and smoking, are found to be associated with diminished NO production [105]. Emerging evidence has also indicated that NO bioavailability dysfunction is implicated in the initiation and development of atherosclerosis [105]. Therefore, improvement of endothelial NO production provides a potential strategy for preventing and managing atherosclerosis.

Abundant evidence has demonstrated the implication of modulating the NO pathway in the effect of natural monoterpenes on endothelial dysfunction and atherosclerosis. NO is synthesized by nitric oxide synthase (NOS) using L-arginine as the substrate [104]. NOS includes different isoforms that play contrasting roles in atherosclerosis, with endothelial NOS (eNOS) being atheroprotective and inducible NOS (iNOS) being pro-atherogenic [105]. Thus, modulating NO by adjusting the eNOS/iNOS ratio is crucial for protecting against endothelial dysfunction and atherosclerosis. Catalpol, a monoterpenoid extracted from the root of the traditional Chinese herb *Rehmanniae radix*, could maintain the balance of endothelial NO by inhibiting the NF-κB/iNOS pathway and by activating the phosphatidylinositol-3-kinase (PI3K)/protein kinase B (Akt)/eNOS pathway. As a result, catalpol improved cell viability, enhanced endothelial integrity, and reduced inflammatory response in advanced glycation end-product (AGE)-treated endothelial cells [94]. Furthermore, two other monoterpenoids, paeoniflorin and eucalyptol, can upregulate SIRT1 expression to regulate the eNOS/iNOS ratio and to protect against endothelial dysfunction [88,93]. Notably, the activation of eNOS by these monoterpenoids depends on either eNOS Ser1177 phosphorylation [94] or upregulation of eNOS expression [88,93]. Oxidative stress plays a crucial role in determining NO bioavailability [104]. During endothelial dysfunction, oxidative stress mediates ONOO^−^ formation and oxidizes eNOS cofactor tetrahydrobiopterin (BH4), causing BH4 deficiency and eNOS uncoupling. Ultimately, it leads to increased ROS generation and reduced NO bioavailability [104]. Perillaldehyde, a major component in essential oil isolated from *Perilla frutescens* that has been used in traditional Chinese medicine, was reported to prevent endothelial dysfunction and to attenuate the growth of atherosclerosis [85]. Mechanically, perillaldehyde alleviated ROS-mediated oxidative stress and rescued BH4 deficiency, thereby promoting eNOS recoupling and improving endothelial dysfunction [85]. Other monoterpenoids, such as citronellal and aucubin, similarly induced eNOS recoupling and ameliorated vascular endothelial injury through an anti-oxidative mechanism [91,98]. Monoterpenes that modulate the endothelial NO pathway are shown in Table 3. 

### 3.4. Attenuation of Endothelial Apoptosis 

Apoptosis is a well-known programmed cell death pathway involved in various physiological and pathological processes. Two molecular pathways are acknowledged to regulate apoptosis: mitochondrial-dependent intrinsic pathway and death receptor-mediated extrinsic pathway. The mitochondrial-dependent apoptosis pathway is induced by cellular damage or stress, which upregulates B cell leukemia/lymphoma 2 (BCL2)-associated agonist of cell death (Bad) and facilitates the insertion of BCL2-associated X protein (Bax) into the mitochondrial outer membrane. This further causes cytochrome c to be released into the cytoplasm, followed by interaction between cytochrome c and apoptotic peptidase activating factor 1 (Apaf-1) to induce apoptosis through caspase activation [106]. The extrinsic apoptotic pathway is initiated by binding extracellular death ligands (such as TNF-α) to their respective cell-surface death receptors. The activation of the death receptors results in the formation of the death-inducing signaling complex mediated by an adaptor protein, thereby triggering caspase activation and the apoptosis process [107]. While apoptosis serves as a fundamental mechanism for physiological homeostasis, uncontrolled apoptosis may lead to cellular dysfunction and death. It has been observed that increased endothelial apoptosis is closely associated with atherosclerosis, and pro-atherosclerotic factors such as ox-LDL, oxidative stress, and low shear stress have been shown to induce vascular endothelial cell apoptosis [106,108]. Therefore, inhibition of endothelial apoptosis may represent a promising strategy to cope with atherosclerosis. 

Accumulating evidence demonstrates the reduction of endothelial apoptosis as one of the mechanisms by which natural monoterpenes protect against endothelial injury and atherosclerosis. The anti-apoptotic effect of monoterpenes involves the modulation of intrinsic and/or extrinsic apoptotic pathways in endothelial cells. For instance, the beneficial impact of geniposide against atherosclerosis and endothelial dysfunction was related to the attenuation of endothelial apoptosis due to upregulation of anti-apoptotic protein Bcl-2, downregulation of pro-apoptotic protein Bax and caspase-3, and maintaining mitochondrial membrane potential [67,68]. Similarly, many other monoterpenes have also been shown to ameliorate endothelial cell apoptosis by modulating the Bcl-2/Bax ratio, caspase-9, caspase-3, the release of cytochrome c, and mitochondrial function [64,66,77,78,83,90,97,109]. All this evidence suggests a mitochondrial-dependent intrinsic pathway as a target in the effect of these monoterpenes. Furthermore, the extrinsic apoptotic pathway is likely implicated in the anti-endothelial apoptotic effect of monoterpenes. Monotropein, an active monoterpenoid isolated from the roots of *Morinda officinalis*, was reported to suppress the phosphorylation of NF-κB and activating protein-1 (AP-1), which further inhibited the expression of pro-inflammatory cytokine TNF-α and reduced cell apoptosis in H_2_O_2_-induced HUVECs. These studies indicated that the protective effect of monotropein on endothelial cells might involve the regulation of the TNF-mediated mitochondrial-independent apoptotic pathway [77]. Similarly, picroside II, the main active constituent of *Picrorhiza scrophulariiflora* belonging to monoterpenoid, was found to decrease the caspase-3 activity and the cleaved caspase-3 protein level to inhibit apoptosis in homocysteine-treated HUVECs, which might also be related to the attenuation of TNF-α production [76]. In addition, another study showed that harpagoside, a monoterpenoid extracted from the traditional Chinese herb *Scrophulariae Radix*, prevented angiotensin II (Ang II)-induced endothelial apoptosis via inactivation of caspase-8/caspase-9/caspase-3, suggesting that harpagoside could exert an anti-apoptosis effect by targeting both intrinsic and extrinsic apoptotic pathways [83]. Monoterpenes that attenuate endothelial apoptosis are listed in Table 4. Notably, various signaling pathways such as PI3K/AKT, SIRT1, Nrf2, NF-κB, MAPK, miR-21/PTEN, and AMPK were involved in the anti-endothelial apoptosis effect of these compounds, most of which are also related with oxidative stress and inflammation (Table 4).

## 4. Natural Monoterpenes Potentially Protect against Atherosclerosis by Targeting Macrophages

Macrophages, the major immune cells in atherosclerotic plaque, are fundamental contributors to the pathophysiology of atherosclerosis. After lipoproteins enter the artery wall, they elicit complex pathological processes involving macrophages, such as monocyte recruitment, macrophage differentiation, engulfment of modified lipoproteins, foam cell formation, and initiation of inflammatory responses, which instigate the progression of atherosclerosis [110]. Therefore, macrophages have become an attractive target for developing therapeutic agents against atherosclerosis.

### 4.1. Reduction of Macrophage-Related Inflammation

Atherosclerosis is a chronic inflammatory disease of the arterial wall where non-resolving inflammation acts as a critical underlying driver in all stages of the disease from its onset to its progression, eventually leading to intimal plaque rupture and atherothrombosis giving rise to myocardial infarction or stroke [6,7]. Large amounts of pro-inflammatory cytokines, chemokines, ROS, and NO are produced by inflammatory macrophages that enter the vascular intima, which drives vascular inflammation in atherosclerosis. Thus, anti-inflammation is considered one of the strategies for dealing with atherosclerosis [111]. 

Studies have been conducted to evaluate the anti-inflammation property of natural monoterpenes in macrophages to determine their potential for atherosclerosis management. Both NF-κB and MAPK inflammatory signaling pathways function as vital nodal points in regulating atherosclerosis, which drive the expression of a large panel of pro-inflammatory genes [112]. As shown in Table 5, most monoterpenes are potent macrophage NF-κB and MAPK inhibitors with concomitant suppression of inflammatory mediators (e.g., TNF-α, IL-1β, IL-6, CC, and CXC chemokines), suggesting that NF-κB and MAPK inactivation is the major mechanism for their anti-inflammatory and potential anti-atherosclerotic effects. For example, geniposide was reported to significantly inhibit the expression of toll-like receptor (TLR)-4 and phosphorylation of NF-κB p65, p38 MAPK, JNK, and ERK in macrophages, paralleling with an attenuated systemic inflammation as shown by lower serum levels of TNF-α, IL-6 and IL-8 in HFD-fed apolipoprotein E (ApoE)^−/−^ mice, which suppressed the development of atherosclerosis [68,113,114]. Transcription factors SIRT1 [115,116,117,118], PPARγ [119,120], and Nrf2 [121,122,123,124,125,126], together with intracellular signaling modulators dual specificity phosphatase 1 (MKP-1) [113] and suppressor of cytokine signaling 3 (SOCS3) [127], were proposed to mediate the inhibitory effect of monoterpenes on NF-κB and/or MAPK activation. 

Inflammasome is an integral component of the innate immune system that responds to pathogens and cellular stress. One of the most widely studied inflammasomes induced upon macrophage activation consists of the sensor NLR family PYRIN domain containing-3 (NLRP3), the adaptor apoptosis-associated speck-like protein containing CARD (ASC), and the effector enzyme caspase-1 [170]. It has been established that activation of the NLRP3 inflammasome requires NF-κB-mediated priming and subsequent inflammasome assembly steps [170]. Upon stimulation, the interaction between NLRP3 and ASC together with the formation of ASC oligomer provides a platform for pro-caspase-1 autocleavage and activation [170]. Subsequently, it triggers the production and release of mature IL-1β and IL-18 [170]. Experimental evidence has shown that NLRP3 inflammasome activation is the major driver of atherosclerosis [171,172,173]. Thus, modulating NLRP3 inflammasome may be beneficial for managing atherosclerosis. Carvacrol, a monoterpene commonly found in the essential oils of the *Laminaceae* family, was proven to inhibit cytokine secretion and NLRP3 inflammasome activation [136]. Treatment with carvacrol-containing active fractions significantly blunted protein interaction between NLRP3 and ASC, suppressed ASC oligomerization, abolished ATP-induced K^+^ efflux, and inhibited the release of caspase-1, IL-1β, and IL-18 in LPS-primed macrophages [136]. Furthermore, this carvacrol-containing extract attenuated LPS-induced NF-κB nuclear translocation. Therefore, these observations indicated that carvacrol inhibits NLRP3 inflammasome activation by interfering in both the priming and inflammasome assembly steps in activated macrophages [136]. Mitochondrial dysfunction plays a critical role in the NLRP3 inflammasome assembly process, characterized by the generation of mitochondrial ROS, a decrease in mitochondrial membrane potential, and the release of mitochondrial DNA [170]. Loganin, a major bioactive monoterpenoid derived from the traditional Chinese herb *Cornus officinalis*, was found to prevent mitochondrial stress as shown by decreased mitochondrial ROS level, increased mitochondrial membrane potential, and reduced mitochondrial DNA such as D-loop [156]. These molecular events ultimately suppressed monosodium uric acid (MSU) crystal-induced NLRP3 inflammasome activation in macrophages [156]. Moreover, Nrf2-mediated cytoprotective signaling pathway regulating intracellular redox homeostasis was found to mediate the inhibition of NLRP3 inflammasome activation upon treatment of monoterpenoids, such as loganin and oleocanthal [122,123,156]. Among others, gentiopicroside, a monoterpenoid separated from a traditional medicine *Gentiana macrophylla*, was reported to inhibit NLRP3 inflammasome activation by suppressing both mRNA and protein expression of NLRP3 inflammasome key components NLRP3, ASC, and caspase-1 in LPS-MSU-induced RAW264.7 macrophages [160]. An inhibitory effect on NLRP3 inflammasome activation has also been reported for other monoterpenes [115,129,135,144,147,159,166].

Monoterpenes that decrease macrophage-related inflammation are shown in Table 5.

### 4.2. Inhibition of Foam Cell Formation

The formation of foam cells in the arterial intima is a hallmark of atherosclerosis. During atherogenesis, circulating monocytes infiltrate into the intima and differentiate into macrophages. Mediated by relevant cell surface receptors, macrophages engorge modified lipoproteins, causing accumulation of cholesteryl ester and free cholesterol in the cells, leading to foam cell formation [110,174]. The resulting cholesterol-laden foam cells can further trigger the release of DAMPs and inflammatory responses, thereby promoting the development of atherosclerosis [174]. Accordingly, the macrophage foam cell is recognized as a target for therapeutic intervention of atherosclerosis [175].

Foam cell formation is finely orchestrated by cholesterol uptake and efflux processes. Engulfment of modified lipoproteins by macrophages to induce foam cell formation depends on a family of pattern recognition receptors known as scavenger receptors, including CD36, SR-A, and LOX-1 [176]. Some monoterpenoids have been shown to target macrophage scavenger receptors to inhibit foam cell formation. Using ox-LDL-treated human macrophages, it was found that oleacein, a monoterpenoid found primarily in olive fruit and leaves, significantly attenuated ox-LDL-induced foam cell formation by decreasing the expression of CD36, SR-A, and LOX-1 [177]. Notably, macrophage apoptosis, a major contributor to the progression of atherosclerotic plaques, was inhibited by oleacein [177,178]. This observation might also be related to the reduced expression of CD36 and SR-A [177,178]. Another two medicinal monoterpenoids, geniposide and albiflorin, showed similar inhibitory effects on ox-LDL-induced foam cell formation by suppressing CD36 or LOX-1 expression and by inhibiting NF-κB- and MAPK-mediated inflammatory response [179,180].

Apart from cholesterol uptake, foam cell formation is also regulated by cholesterol efflux from macrophages. Diminished efflux of intracellular cholesterol to extracellular receptors facilitates the formation of foam cells. It has been known that macrophage cholesterol efflux is mediated by ATP-binding cassette transporters ABCA1, ABCG1, and SR-BI [176]. Targeting ABCA1, ABCG1, and SR-BI to promote cholesterol efflux represents a potential strategy to inhibit foam cell formation and atherosclerosis. Liver X receptors (LXRs), members of the nuclear receptor superfamily, play a pivotal role in RCT by which accumulated cholesterol in the peripheral tissue is transported to the liver for excretion [181]. As ligand-activated transcription factors, LXRs regulate cellular cholesterol homeostasis by promoting the expression of genes related to cholesterol efflux, such as ABCA1 and ABCG1 [181]. Previous studies reported that eucalyptol, a principal monoterpene in plant-derived essential oils, significantly stimulated the expression of LXRα and LXRβ followed by induction of their target genes ABCA1 and ABCG1 expression in macrophages, which in turn promoted cholesterol efflux and suppressed ox-LDL-induced foam cell formation [182,183]. Eucalyptol decreased the expression of LXRα and lipogenesis-related genes in hepatocytes, resulting in attenuated hepatic lipid accumulation [183]. Given that many synthetic LXR agonists cause hepatic lipogenesis and hypertriglyceridemia, eucalyptol can serve as a potent pharmaceutical agent against atherosclerosis by activating LXRs in macrophages without inducing the side effects of hepatic lipogenesis and steatosis [183]. Moreover, a recent study showed that geniposide attenuated foam cell formation and atherosclerosis by inhibiting lipid uptake and by promoting cholesterol efflux [184]. Mechanistically, it was found that this natural monoterpenoid could reduce the expression of SR-A. At the same time, it promotes the expression of ABCA1 and SR-BI in both lysophosphatidic acid (LPA)-induced macrophages and HFD-fed ApoE^−/−^ mice, leading to attenuated foam cell formation and atherosclerosis [184]. Notably, modulation of SR-A, ABCA1, and SR-BI by geniposide was proposed to be mediated by p38 MAPK and AKT signaling pathways [184]. 

Monoterpenes that inhibit foam cell formation have been listed in Table 6. 

### 4.3. Induction of Macrophage Autophagy

Autophagy is an intracellular lysosomal degradation process that plays a housekeeping role in clearing dysfunctional proteins, damaged organelles, and intracellular pathogens. Key factors involved in autophagy regulation have been identified, including mTOR, AMPK, and Beclin1 [195]. As the central players in the pathogenesis of atherosclerosis, macrophages have been demonstrated to undergo a progressive autophagy dysfunction in the developing atherosclerotic lesion, resulting in hyperactivation of inflammation, increase in cell death, and reductions in lipophagy and cholesterol efflux [196]. Growing evidence suggests that induction of autophagy in macrophages can exert an atheroprotective effect by eliminating long-lived/damaged intracellular material, attenuating excessive inflammation and cell death, promoting macrophage efferocytosis, and accelerating cholesterol efflux from foam cells, eventually inhibiting plaque development and instability [195]. Thus, activation of macrophage autophagy is expected to be beneficial for preventing atherosclerosis [195]. 

Geniposide, a bioactive monoterpenoid isolated from *Gardenia jasminoides*, has been reported to be atheroprotective, as shown by improved serum lipid profile, decreased aortic lipid deposition, reduced macrophage-mediated inflammation, attenuated apoptosis, and smaller size of atherosclerotic plaques [68,184]. A recent study provided evidence that geniposide could inhibit the progression of atherosclerosis by promoting macrophage autophagy [188]. Mechanistic studies further indicated that geniposide inhibited the expression of triggering receptor expressed on myeloid cells 2 (TREM2) and the phosphorylation of mTOR, which in turn enhanced macrophage autophagy [188]. These results suggest TREM2/mTOR signaling as an intracellular target in the protective effect of geniposide on macrophage autophagy and atherosclerosis [188]. Paeoniflorin, a monoterpenoid in the traditional Chinese herb *Paeonia lactiflora*, was previously reported to promote autophagy in ox-LDL-induced HUVECs, as demonstrated by increased microtubule-associated protein 1 light chain 3-II (LC3-II)/LC3-I ratio and decreased p62 protein level [64]. Consistently, an increased autophagic response was also observed in IL-4-induced pro-fibrotic macrophages in the presence of paeoniflorin-containing compound combination, which was mediated by attenuation of AKT/mTOR signaling [197]. However, whether paeoniflorin can regulate macrophage autophagy under atherogenic conditions and exert an atheroprotective effect remains elusive and is worth further investigation.

Prevention of defective macrophage autophagy in plaques is considered a novel strategy to protect against atherosclerosis [196]. Although emerging data reveal that natural products are promising in stimulating autophagy [195], our understanding of the role of monoterpenes in the regulation of macrophage autophagy in atherosclerosis is somewhat limited, and it thus requires further studies. Since autophagy is a double-edged sword in cardiovascular disease [79], more selective and precise manipulation of autophagy is also needed to achieve better therapeutic outcomes. 

### 4.4. Enhancement of M2 Macrophage Polarization

As important players in the innate immune system, macrophages are characterized by remarkable phenotypic and functional plasticity. Based on the dynamic microenvironmental stimuli in the atherosclerotic plaque, macrophages can be polarized into subsets with distinct functional properties, which include two well-known macrophage phenotypes: classically activated (M1) macrophages and alternatively activated (M2) macrophages [198]. M1 macrophages produce a variety of inflammatory mediators, which exacerbate the development of atherosclerosis [199]. In contrast, M2 macrophages exert a protective effect on atherosclerosis by releasing anti-inflammatory factors and promoting tissue reparative processes to inhibit plaque growth and enhance plaque stability [198,199]. Thus, modification of plaque macrophage polarization by enhancing M2 macrophage phenotype is a potential therapeutic strategy to ameliorate atherosclerosis.

Several studies have shown natural monoterpenes’ capacity to promote M2 macrophage polarization. Signal transducer and activator of transcription 3 (STAT3) and STAT6 are recognized as key signaling molecules responsible for M2 macrophage polarization, which are mediated by IL-4/13 and IL-10 signaling, respectively [200]. Oleacein, a monoterpenoid in extra-virgin olive oil, was reported to upregulate the expression of CD163 (a biomarker of M2-type macrophages) and increase the phosphorylation of Janus kinase (JAK1)/JAK2 and STAT3 in ox-LDL-induced human macrophages, suggesting that oleacein promotes the polarization of anti-inflammatory M2-type macrophages via the JAK/STAT3 pathway [177,194]. An ex vivo study using human carotid plaques obtained from patients with transient ischemic attacks revealed that oleacein could suppress plaque instability by attenuating related biomarkers such as HMGB1 and matrix metalloproteinase-9 (MMP-9) [201]. Therefore, it is reasoned that oleacein-mediated M2 macrophage polarization contributed to the protective effect of oleacein on atherosclerotic plaque vulnerability. Compound edaravone injection is an approved drug for acute ischemic stroke that consists of edaravone and borneol (a monoterpene) [186]. Pharmacological studies revealed that although borneol alone showed no apparent effect on modulating macrophage phenotype, it could reinforce the efficacy of edaravone in promoting M2 macrophage polarization, as indicated by increased expression of CD206, arginase-1 (Arg-1) and IL-10 [186]. Mechanistically, this pharmacological action of borneol was related to the activation of the JAK/STAT3 signaling pathway [186]. However, other monoterpenoids, such as genipin and paeoniflorin, were shown to enhance M2 macrophage polarization by activating STAT6 [189,191]. Estrogen deficiency is a known risk factor for atherosclerosis in postmenopausal women [190]. Catalpol, a monoterpenoid in the traditional Chinese herb *Rehmanniae radix*, was demonstrated to attenuate atherosclerotic lesions in a mouse model of postmenopausal atherosclerosis by accelerating M2 macrophage polarization [190]. The underlying mechanism was related to the molecular interaction between catalpol and the estrogen receptor (ER) leading to the activation of ERα, suggesting that catalpol is a potent therapeutic agent against postmenopausal atherosclerosis [190]. Monoterpenes involved in promoting M2 macrophage polarization are shown in Table 6. 

Notably, other macrophage populations, in addition to M1/M2 macrophages, have been indicated in atherosclerotic plaque [202,203]. For example, M2-type macrophages are reported to be divided into M2a, M2b, M2c, and M2d subsets with distinct functions in atherosclerosis [203]. However, there is still a significant knowledge gap regarding macrophage heterogeneity and their roles in atherosclerosis, as well as the effects of monoterpenes on these macrophage sub-populations, which warrant further investigations. 

## 5. Natural Monoterpenes Potentially Protect against Atherosclerosis by Targeting Vascular Smooth Muscle Cells 

Vascular smooth muscle cell (VSMC) is a constitutive part of the blood vessel and plays essential roles in regulating arterial physiology and pathology. Under physiological conditions, VSMCs are characterized by the expression of a range of genes required for their differentiated and contractile phenotype [204]. In response to vascular injury or atherosclerosis, however, VSMCs undergo phenotypic switching, which refers to the de-differentiation of the contractile phenotype to an alternative non-contractile VSMC phenotype [204]. These phenotypically modulated VSMCs typically exhibit reduced expression of contractile markers and increased capacity for cell proliferation, migration, and secretion, which are critically involved in the pathological process of atherogenesis [205,206]. Within the atherosclerotic plaque, the modulated VSMCs can adopt a variety of phenotypes with different effects on atherosclerosis, some of which have been considered to be detrimental such as macrophage-like and foam cell-like VSMCs [205,206]. Remarkably, many human genomic loci identified to be associated with the risk of coronary artery disease are related to VSMC phenotypic modulation, suggesting that targeting the phenotypic transition of VSMCs may help to attenuate plaque burden and promote plaque stability [206]. 

Enhanced proliferation and migration are hallmarks of VSMC phenotypic switching that underlies many vascular diseases. A number of natural monoterpenes have been proven to inhibit abnormal proliferation and migration of VSMCs, indicating their potential pharmacological application to treat vascular disorders. The MAPK family, including p38 MAPK, JNK, and ERK is a class of serine/threonine protein kinases essential for cell proliferation and migration [207]. As shown in Table 7, most monoterpenes could target MAPK signaling to suppress aberrant VSMC proliferation and migration. For instance, linalool, a monoterpene in many aromatic medicinal plants, inhibited Ang II-induced VSMC proliferation and migration by suppressing cholinergic receptor muscarinic 3 (CHRM3)-dependent activation of p38 MAPK, JNK, and ERK signaling [208]. However, many other monoterpenes selectively inhibit MAPKs in stimulated VSMCs. Carvacrol, a monoterpene found in plant essential oils and commonly used as a food additive and flavoring agent, was shown to inhibit p38 MAPK and ERK activation, thereby attenuating platelet-derived growth factor-BB (PDGF-BB)-induced VSMC proliferation and migration [209]. Furthermore, carvacrol was observed to decrease ROS generation [209] markedly. Since ROS has been reported to promote VSMC proliferation and migration through MAPK signaling [210], these observations indicated that the anti-proliferative and anti-migratory effects of carvacrol on PDGF-BB-induced VSMCs were achieved by the regulation of the ROS-mediated p38 MAPK/ERK pathway. Consistently, thymoquinone, a monoterpene derived from the medicinal plant *Nigella sativa*, could activate the AMPK/PPARγ/PGC-1α pathway to downregulate ROS production and further attenuate p38 MAPK-mediated VSMC proliferation and migration [211,212]. Similarly, the monoterpenoid paeoniflorin was also reported to suppress ox-LDL- and PDGF-BB-induced proliferation and migration of VSMCs through ROS-mediated p38 MAPK and ERK signaling pathways [192,213]. Notably, the reduction of ROS by paeoniflorin was related to the increased expression of heme oxygenase-1 (HO-1), which is an essential cytoprotective gene regulating intracellular redox homeostasis [192]. In addition to MAPKs, AKT signaling is a classic pathway involved in cell proliferation and migration. The monoterpenoid, genipin, was shown to target not only HO-1/ERK but also AKT signaling to inhibit TNF-α-induced VSMC proliferation and migration [214]. Other cell proliferation and migration-related signaling molecules, such as phospholipase C-γ1 (PLC-γ1) and STAT3, were also reported to be targeted by monoterpenoids hinokitiol [215] and plumericin [216]. Monoterpenes that regulate VSMC proliferation and migration are listed in Table 7.

Although the above-mentioned monoterpenes can inhibit abnormal VSMC proliferation and migration, whether these compounds can achieve an atheroprotective effect through modulating VSMC phenotypic switching is unclear. VSMC phenotypic switching is a rather complex process in atherosclerosis. Depending on various environmental cues, VSMCs exhibit different phenotypes that may have either beneficial or maladaptive effects on atherosclerosis. However, the exact role and the regulatory mechanism of each VSMC phenotype in atherogenesis remain largely elusive. Therefore, much work remains to be conducted regarding VSMC phenotypic switching in order to perform a better pharmacological intervention to stimulate atheroprotective VSMC phenotypes and to eliminate detrimental VSMC phenotypes.

## 6. Natural Monoterpenes Potentially Protect against Atherosclerosis by Targeting Dendritic Cells 

Atherosclerosis is characterized by chronic arterial inflammation involving innate and adaptive immune responses. Dendritic cells, a class of hematopoietic cells, function as the necessary connection between innate and adaptive immunity. Throughout atherogenesis, an increased accumulation of vascular dendritic cells has been demonstrated in atherosclerotic lesions, indicating their involvement in the development of atherosclerosis [223]. It has been shown that dendritic cells are highly heterogenous with multifaced roles in the pathophysiology of atherosclerosis, which include lipid uptake, efferocytosis, antigen presentation, T cell activation, and the production of cytokines/chemokines [224,225]. Targeting dendritic cells based on their immunoregulation property has provided encouraging results in attenuating experimental atherosclerosis, indicating the promise of its clinical application for atherosclerosis treatment [223]. 

Oral administration of geniposide, a monoterpenoid isolated from the traditional Chinese herb *Gardenia jasminoides*, was shown to ameliorate the development of atherosclerosis in HFD-fed ApoE^−/−^ mice [222]. It was also demonstrated that geniposide decreased dendritic cell maturation in the bone marrow and infiltrated the atherosclerotic lesions [222]. As we know, upon maturation, the expression of biomarkers of dendritic cells is increased, such as CD11c, CD83, CD80, and CD86, which regulates antigen presentation and induction of T cell-related adaptive immunity and thus facilitates the development of a pro-atherogenic phenotype [223,225]. Nevertheless, geniposide treatment resulted in decreased expression of CD11c, CD83, CD80, and CD86 in ApoE^−/−^ mice, indicating that geniposide might inhibit dendritic cell maturation and suppress the development of pro-atherogenic effector T cells, thus alleviating atherogenesis [222]. Moreover, dendritic cell-derived IL-12 decreased after geniposide treatment [222], an essential pro-atherogenic cytokine that activates T cells and induces the generation of pro-inflammatory T-helper 1 (Th1) cells [225]. Taken together, the atheroprotective role of geniposide may be attributed to the inhibition of both dendritic cell maturation and pro-inflammatory cytokine production. Similarly, the monoterpenoid paeoniflorin was demonstrated to suppress dendritic cell maturation and subsequent Th17 cell differentiation, which was related to the attenuation of NF-κB and JNK signaling pathways [219]. Another study reported that paeoniflorin could target TGF-β signaling to induce a regulatory dendritic cell subtype characterized by high expression of CD11b/c and low expression of co-stimulatory molecules [220]. This regulatory dendritic cell showed a striking stimulative effect on anti-inflammatory regulatory T (Treg) cells that protect against atherosclerosis, indicating the potential of paeoniflorin as an immunosuppressive and atheroprotective agent [220]. Other monoterpenes, such as carvacrol [217], thymol [217], and thymoquinone [218], exhibit similar inhibitory effects on dendritic cell maturation and function (Table 7).

## 7. Conclusions and Future Perspectives

**Figure 2:**
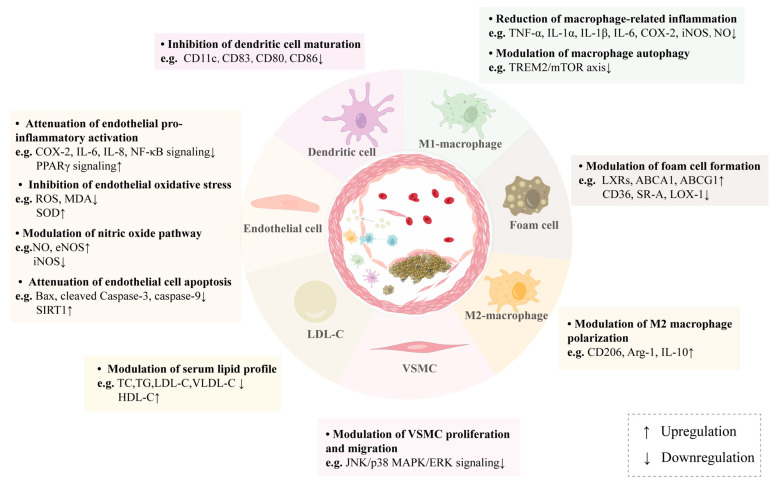
Pharmacological actions of natural monoterpenes on atherosclerosis. Atheroprotective monoterpenes can target vascular cells involved in atherosclerosis, including endothelial cells, monocytes/macrophages, VSMCs, and dendritic cells. Moreover, they modulate various atherogenesis-related pathological processes, such as hyperlipidemia, inflammation, oxidative stress, apoptosis, NO bioavailability, foam cell formation, autophagy, and abnormal cell proliferation and migration. This figure schematizes atherosclerotic plaque formation in the middle panel, with various target cell types (or pro-atherogenic factor) and relevant pharmacological actions of antiatherogenic monoterpenes shown around the outer margins.

**Figure 3:**
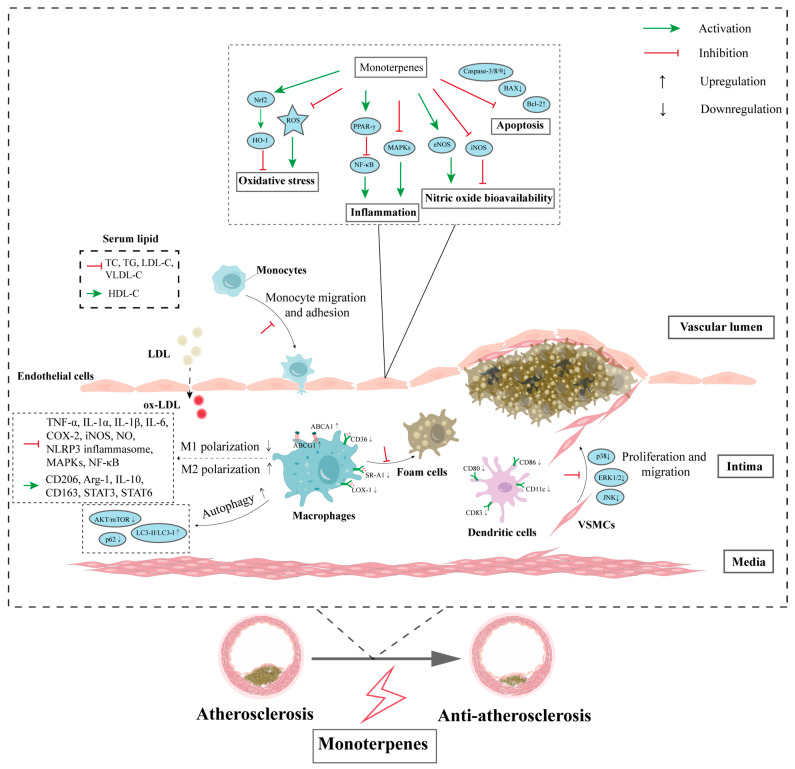
The cellular and molecular mechanisms of natural monoterpenes in the development of atherosclerosis. Monoterpenes target various pro-atherogenic factors and intracellular molecular pathways in atherogenesis-related cell types, thus leading to the attenuation of atherosclerosis.

Monoterpenes, a large class of naturally occurring compounds in many aromatic and medicinal plants, have drawn increasing attention due to their therapeutic potential in cardiovascular disease management. Herein, we reviewed published studies concerning natural monoterpenes that have potential atheroprotective properties and highlighted their underlying mechanisms at the cellular and molecular levels. In summary, the cellular targets of atheroprotective monoterpenes are diverse. Monoterpenes regulate the biological functions of endothelial cells, monocytes/macrophages, VSMCs, and dendritic cells through various pharmacological mechanisms, including serum lipid-lowering properties, anti-inflammatory effects, anti-oxidative effects, anti-apoptosis effects, inhibition of foam cell formation, suppression of ER stress, activation of autophagy, and attenuation of VSMC proliferation and migration (Figure 2). Moreover, molecular targets mediating the atheroprotective effect of monoterpenes mentioned in this review include SIRT1, PPARγ, Nrf2, STAT, NF-κB, MAPK, PI3K/AKT, AMPK, and mTOR (Figure 3).

Despite significant research progress on the effects and mechanisms of natural monoterpenes on atherosclerosis, developing novel therapeutic agents from these compounds as effective therapies for atherosclerosis requires more detailed investigations. First, many monoterpenes demonstrate low bioavailability upon oral administration, which significantly dampens their pharmacological actions in vivo. Therefore, it is imperative to develop alternative strategies for drug delivery to improve their bioavailability and pharmacokinetic properties. Currently, nanotechnology and structural modification techniques have emerged as promising approaches to facilitate in vivo drug delivery. Remarkably, preclinical studies using nanostructured lipid carriers for eucalyptol, thymoquinone, and oleuropein, three monoterpenoids with poor bioavailability due to low aqueous solubility and stability, have shown encouraging results with significantly improved bioavailability and efficacy in vivo [92,226,227]. In addition, structural alterations of swertiamarin, a monoterpenoid with a low plasma half-life, resulted in two new derivatives of swertiamarin with enhanced stability in the circulation and thus may accelerate its bioavailability [228]. However, these approaches that aim to improve the bioavailability of monoterpenes should be further tested on human subjects with atherosclerosis to evaluate their antiatherogenic efficacy. Second, although a number of monoterpenes have been demonstrated to be atheroprotective, few studies mention adverse effects or toxicity. Therefore, comprehensive toxicity studies should be carried out to investigate the safety of these compounds for drug development. Concurrently, given that there are not many data available on the pharmacokinetics of monoterpenes, it is necessary to perform comprehensive absorption–distribution–metabolism–excretion investigations in vivo to assess the effectiveness and safety of candidate monoterpenes. Third, while a large proportion of pharmacological studies regarding antiatherogenic monoterpenes have been conducted at the levels of experimental animals and cultured cells, further clinical studies are required to examine the therapeutic effects of candidate monoterpenes on atherosclerosis. In this regard, randomized, double-blind, placebo-controlled clinical trials with large samples are warranted for rigorous evaluation of the potency of clinical translation.

Notably, as shown in this review, the potential antiatherogenic effects of most of the monoterpenes may be mediated by multiple mechanisms involving different cell types and molecular pathways, suggesting their pleiotropic or multitargeted pharmacological properties. Since atherosclerosis has been established as a multifaceted vascular disease, the pleiotropic pharmacological actions of monoterpenes may hold great promise for the therapeutic intervention of atherosclerosis by targeting multiple pathological processes in atherogenesis. However, many current data are based on in vitro studies. Thus, more in vivo investigations using animal models of atherosclerosis should be conducted to confirm these in vitro findings. Additionally, it is tempting to expect that the combination of different monoterpenoids or other bioactive herbal ingredients may further improve the efficacy in treating atherosclerosis as a result of their potential synergistic effects. For example, a patented drug combination used for the management of atherosclerosis (patent no. CN201810425740.8) consists of a monoterpenoid geniposide and the other natural compound, notoginsenoside R1 [68]. It has been reported that this drug combination exhibits better efficacy in atherosclerosis alleviation than either geniposide or notoginsenoside R1 alone [68]. Therefore, comprehensive and rigorous analysis of the effectiveness and mechanisms of each monoterpene alone and in combination is of enormous significance and should be strengthened in future studies.

## Figures and Tables

**Table 1:** Modulation of serum lipid profile.

Monoterpene	Experimental Model	Efficacy	Target/Pathway	Refs.
Linalool	C57BL/6J mice fed with HFD	Lowers TC, TG, and LDL-C, and improves atherogenic index	Suppresses SREBP-2-mediated HMG-CoA reductase expression and induces ubiquitin-dependent proteolysis of the HMG-CoA reductase	[24]
Amarogentin	C57BL/6J mouse model of diabetes	Attenuates neointimal thickening, and collagen and lipid deposition in the aorta, lowers TC, TG, LDL-C, and VLDL-C, and increases HDL-C	Induces phosphorylation of AMPK	[26]
Oleuropein	Wistar rats fed with a cholesterol rich diet	Decreases TC and TG, and elevates HDL-C	Suppresses lipid synthesis and promotes fatty acid oxidation mediated by AMPK	[27]
Aucubin	C57BL/6J mice treated with tyloxapol	Downregulates TC, TG, LDL-C and VLDL-C, and increases HDL-C	Activates AMPK and Nrf2, and promotes the expression of PPARα and PPARγ	[28]
Thymoquinone	SD rats fed with HFD	Decreases TC and LDL-C levels	Downregulates gene expression of HMG-CoA reductase and upregulates the expression LDLR	[30]
Geniposide	C57BL/6J mice and ApoE^−/−^ mice fed with HFD	Suppresses atherosclerotic plaque progression, reduces serum TC and TG, and attenuates hepatic lipid deposition	Regulates FXR-mediated liver-gut crosstalk of bile acids	[31]
Swertiamarin	SD rats fed with a cholesterol rich diet	Lowers TC, TG, LDL-C, VLDL-C, and atherogenic index	Inhibits HMG-CoA reductase activity and enhances the fecal bile acid and total sterols excretion	[34]
Eucalyptol	Wistar rat model of diabetic-atherosclerosis	Prevents the formation of the atheromatous lesions, reduces TC, TG, and LDL-C, and upregulates HDL-C	Not shown	[35]
Geraniol	Syrian hamsters fed with an atherogenic diet	Decreases TC, TG, free fatty acids, phospholipids, LDL-C, and VLDL-C, upregulates HDL-C, and lowers atherogenic index	Inhibits HMG-CoA reductase and suppresses lipogenesis	[36]
Thymol	New Zealand white rabbits fed with HFD	Decreases TC, TG, and LDL-C, and elevates HDL-C	Not shown	[37]
Limonene	Wistar rats fed with an atherogenic diet	Reduces TG, TC, VLDL-C, LDL-C and non-HDL-C levels, and elevates HDL-C/LDL-C, HDL-C/TC and HDL-C/TG	Reduces HMG-CoA reductase activity	[38]
p-Cymene	Wistar rat model of diabetes	Improves TC, TG, LDL-C, VLDL-C, and HDL-C	Suppresses AKT/mTOR signaling	[39]
Safranal	Wistar rat model of diabetes	Decreases total lipids, TC, TG, and LDL-C, and elevates HDL-C	Not shown	[40]
Genipin	C57BL/6J mice fed with HFD; mouse primary hepatocytes treated with free fatty acids	Antagonizes HFD-induced hyperlipidemia and hepatic lipid accumulation	Regulates miR-142a-5p/SREBP-1c axis	[41]
Catalpol	New Zealand white rabbits fed with HFD and EA. hy926 cells treated with ox-LDL	Attenuates atherosclerotic lesions, decreases TC, TG, and LDL-C, and increases HDL-C	Promotes Nrf2/HO-1-mediated anti-oxidative stress and inhibits NF-κB-mediated inflammation	[42]
Paeoniflorin	C57BL/6J mice fed with HFD	Exerts an antagonistic effect on hyperlipidemia and lipid ectopic deposition	Lowers the lipid synthesis pathway, promotes fatty acid oxidation and increases cholesterol output	[43]
Loganic acid	New Zealand rabbits fed with a cholesterol rich diet	Decreases intima thickness and intima/media ratio in the thoracic aorta, lowers TG and ox-LDL, and increases HDL-C	Promotes the expression of PPARα and PPARγ	[44]
Loganin	C57BLKS/J type 2 diabetic db/db mice	Reduces TG and LDL-C/VLDL-C, and increases HDL-C	Suppresses gene expressions related to lipid synthesis and adjusts the abnormal expression of PPARα and SREBPs in the nucleus	[45]
Oleacein	C57BL/6J mice fed with HFD	Reduces TC, TG, and LDL-C	Downregulates the expression of FAS, SREBP-1, and phospho-ERK	[46]
Gentiopicroside	C57BL/6J mice treated with tyloxapol and HepG2 cells treated with free fatty acid	Reduces TC and TG	Regulates Nrf2-mediated PPARα activation and SREBP-1c inactivation	[47]

**Table 2:** Attenuation of endothelial pro-inflammatory activation.

Monoterpene	Experimental Model	Efficacy	Target/Pathway	Refs.
Eucalyptol	HUVECs treated with LPS	Attenuates adhesion molecules and pro-inflammatory cytokines	Regulates PPAR-γ dependent modulation of IκBα/NF- κB signaling	[52]
Citral	HUVECs treated with LPS	Suppresses the adhesion of neutrophils to HUVECs, and decreases adhesion molecules and pro-inflammatory cytokines	Regulates PPAR-γ dependent modulation of IκBα/NF- κB signaling	[53]
Citronellol	Bovine arterial endothelial cells treated with LPS	Suppresses LPS-induced COX-2 expression and attenuates vascular endothelial inflammation	Activates PPARγ signaling	[54]
Genipin	HUVECs treated with TNF-α	Attenuates the adhesion of U937 monocytic cells to HUVECs and ameliorates adhesion molecules	Induces the expression of PPAR-γ	[55]
Cornuside	HUVECs treated with TNF-α	Attenuates pro-inflammatory mediator and adhesion molecules	Suppresses NF-κB signaling	[63]
	HUVECs treated with LPS or HMGB1	Inhibits endothelial permeability, decreases pro-inflammatory mediators, and reduces adhesion events	Modulates SIRT1/HMGB1-mediated NF-κB, ERK and p38 MAPK signaling	[57]
Paeoniflorin	HUVECs treated with LPS	Suppresses the expression of adhesion molecules and pro-inflammatory cytokines	Decreases the activation of IκBα/NF- κB, p38 MAPK and JNK pathway	[61]
	HUVECs treated with ox-LDL	Attenuates adhesion molecule expression	Enhances autophagy via upregulation of SIRT1	[64]
	HUVECs treated with LPC	Suppresses LPC-induced inflammatory factor production	Inhibits the HMGB1-RAGE/TLR-2/TLR-4-NF-κB pathway	[58]
	HUVECs treated with LPS	Suppresses pro-inflammatory cytokines and chemokine	Inhibits ER stress-dependent IRE1α /NF-κB signaling	[65]
Catalpol	Human aortic endothelial cells treated with homocysteine	Inhibits the expression of adhesion molecules and chemokine	Suppresses ER stress and NF-κB signaling	[66]
Geniposide	HUVECs treated with LPS	Inhibits LPS-induced expression of IL-6 and IL-8, and suppresses U937 monocyte adhesion to HUVECs	Attenuates IκBα/NF-κB, p38 MAPK and ERK signaling	[62]
	HUVECs treated with a high level of glucose	Suppresses the adhesion of monocytes to HUVECs and reduces adhesion molecules	Attenuates ROS/NF-κB signaling	[60]
	HUVECs treated with ox-LDL	Decreases the production of pro-inflammatory cytokines	Enhances the miR-21/PTEN pathway	[67]
	ApoE^−/−^ mice fed with HFD and HUVECs treated with H_2_O_2_	Suppresses atherosclerosis and inhibits endothelial inflammation	Modulates AMPK/mTOR/Nrf2 signaling pathway	[68]
Bornyl acetate	HUVECs treated with ox-LDL	Suppresses the attachment of THP-1 monocytes to HUVECs, and ameliorates adhesion molecules and pro-inflammatory cytokines	Mitigates the activation of the IκBα/NF-κB signaling pathway	[69]
Carvacrol	C57BLKS/J type 2 diabetic db/db mice and HUVECs treated with a high level of glucose	Alleviates the histological abnormalities of the abdominal aorta and reduces vascular inflammation	Reduces the activation of the TLR4/NF-κB signaling	[70]
Hinokitiol	SEVC4-10 endothelial cells treated with culture medium of LPS-stimulated RAW 264.7 cell	Inhibits pro-inflammatory cytokine-induced adhesion molecules	Not shown	[71]
Thymoquinone	HUVECs treated with LPS	Suppresses pro-inflammatory cytokines and chemokine	Modulates TET2/NLRP3 inflammasome axis	[72]
Amarogentin	C57BL/6J diabetic mice, and EAhy926 cells or HUVECs treated with TNF-α	Exerts anti-atherosclerotic effects and inhibits endothelial inflammation and the adherence of THP-1 monocytes onto HUVECs	Regulates AMPK/NF-κB pathway	[26]
Oleuropein	HUVECs treated with LPS, TNF-α or PMA	Suppresses the adhesion of monocytes to HUVECs and reduces the adhesion molecule VCAM-1	Inhibits the binding of NF-κB, AP-1, and possibly GATA to the promoter of VCAM-1	[73]
Oleacein	HUVECs treated with LPS or TNF-α	Suppresses the adhesion of monocytes to HUVECs and reduces adhesion molecules and pro-inflammatory chemokine	Inhibits NF-κB-mediated CCL2 expression	[74]
Plumericin	HUVECs treated with TNF-α	Attenuates adhesion molecule expression	Regulates IKK/IκB/NF-κB pathway	[75]
Picroside II	HUVECs treated with homocysteine	Reduces the production of inflammatory mediators	Modulates the SIRT1/LOX-1/NF-κB signaling pathway	[76]
Monotropein	HUVECs treated with H_2_O_2_	Alleviates the inflammatory response of HUVECs	Attenuates NF-κB/AP-1 signaling	[77]
Albiflorin	HUVECs treated with ox-LDL	Alleviates the production of pro-inflammatory cytokines	Blocks IRAK1/TAK1 pathway	[78]

**Table 3:** Inhibition of endothelial oxidative stress and modulation of nitric oxide pathway.

Monoterpene	Experimental Model	Efficacy	Target/Pathway	Refs.
Geraniol	C57BL/6J mice fed with HFD and HUVECs treated with palmitic acid	Protects against HFD-induced endothelial dysfunction	Decreases the expression of NOX-2 to suppress ROS production	[81]
	Syrian hamsters fed with an atherogenic diet	Attenuates endothelial dysfunction and prevents tissue oxidative injury	Increases the expression of Nrf2, inhibits lipid peroxidation and reduces antioxidant enzymes (SOD, CAT, GPx, and GR)	[87]
Paeoniflorin	HUVECs treated with H_2_O_2_	Attenuates H_2_O_2_-induced endothelial cell damage	Increases the expression of SIRT1 and modulate balance between eNOS/iNOS	[88]
	HUVECs treated with H_2_O_2_	Suppresses H_2_O_2_-induced oxidative stress	Scavenges intracellular ROS, and rescues abnormalities of MDA, SOD and GSH-Px	[89]
	HUVECs treated with TBHP	Suppresses TBHP-induced oxidative damage	Activates Nrf2/HO-1 signaling to reduce ROS level and increase activities of CAT, GPx, and SOD	[90]
	HUVECs treated with AOPPs	Protects against AOPP-induced oxidative damage	Suppresses ROS generation through the inhibition of RAGE-NOX2/NOX4	[82]
Harpagoside	bEnd.3 endothelial cells treated with Ang II	Inhibits Ang II-induced oxidative stress	Decreases NOX2/NOX4/COX-2/ROS and lipid peroxidation level	[83]
Thymoquinone	Rabbit aortic rings treated with pyrogallol	Exerts antioxidant capacity, increases NO production, and improves pyrogallol-induced endothelial dysfunction	Reduces lipid peroxidation and enhances activity or content of SOD and GSH	[84]
Perillaldehyde	Rats and ApoE^−/−^ mice with HFD or plus balloon injury and HUVECs treated with ox-LDL	Suppresses oxidative stress to improve endothelial dysfunction with increased NO generation and inhibits atherosclerosis	Increases endogenous BH4 generation, rescues abnormalities of ROS, MDA and SOD, and elevates consequent eNOS recoupling	[85]
Citronellal	SD rats fed with HFD plus balloon injury	Suppresses oxidative stress, increases NO production, and prevents endothelial dysfunction and the progression of atherosclerosis	Downregulates the expression of NHE1 and rescues abnormalities of MDA and SOD activity	[86]
	SD rats fed with HFD plus streptozotocin (STZ) administration, and HUVECs treated with a high level of glucose	Alleviates oxidative stress, increases NO production, and improves high glucose-induced endothelial injury	Induction of S1P/S1P1 signaling, increases eNOS expression, recouples eNOS, and rescues abnormalities of NOx, ROS, MDA and SOD and other anti-oxidant enzymes	[91]
Geniposide	HUVECs treated with ox-LDL	Inhibits ox-LDL-induced oxidative stress	Modulates the miR-21/PTEN/NOX2 pathway, and rescues abnormalities of ROS, MDA, SOD, GSH-Px, and CAT	[67]
	ApoE^−/−^ mice fed with HFD and HUVECs treated with H_2_O_2_	Suppresses atherosclerosis and inhibits ox-LDL-induced oxidative stress	Modulates AMPK/mTOR/Nrf2 pathway and rescues abnormalities of NOX2, ROS, MDA, GSH, and SOD	[68]
Monotropein	HUVECs treated with H_2_O_2_	Ameliorates H_2_O_2_-mediated oxidative injury	Attenuates NF-κB/AP-1 signaling and rescues abnormalities of MDA, SOD, and GSH-Px	[77]
Eucalyptol	HUVECs treated with a high level of glucose	Protects against high glucose-induced vascular endothelial injury	Modulation of Keap1/Nrf2/HO-1 signaling to reduce ROS generation	[92]
	HUVECs treated with LPS	Sustains the balance of endothelial NO and ameliorates LPS-induced HUVEC injury	Suppresses NF-κB signaling to reduce iNOS-derived NO, and recovers eNOS-derived NO to the normal level	[93]
Catalpol	New Zealand white rabbits fed with HFD and EA.hy926 cells treated with ox-LDL	Exerts beneficial effects on atherosclerosis progression, oxidative stress and inflammation	Rescues abnormalities of MDA, SOD and GSH-Px, and induces the activation of Nrf2/HO-1 axis in HUVECs	[42]
	Mouse glomerular endothelial cells treated with AGE	Ameliorates AGEs-induced endothelial dysfunction	Inhibits the NF-κB/iNOS pathway and activates the PI3K/AKT/eNOS pathway	[94]
	Human aortic endothelial cells treated with homocysteine	Inhibits homocysteine-induced oxidative damage	Decreases NOX4/ROS signaling and rescues abnormalities of MDA and GSH	[66]
Citral	HUVECs treated with H_2_O_2_	Exerts antioxidant capacity and protects against oxidative damage induced by H_2_O_2_ in HUVECs	Reduces hydroperoxide levels and elevates total antioxidant activity	[95]
Genipin	HUVECs treated with thrombin	Inhibits thrombin-induced VWF release and P-selectin translocation in HUVECs	Activates eNOS phosphorylation, promotes enzyme activation and increases NO production	[96]
Safranal	Bovine aortic endothelial cells treated with H_2_O_2_	Suppresses H_2_O_2_-induced oxidative stress	Decreases ROS production	[97]
Amarogentin	C57BL/6J diabetic mice and EA.hy926 cells or HUVECs treated with TNF-α	Exerts anti-atherosclerotic effects and inhibits endothelial dysfunction	Regulates AMPK/eNOS pathway	[26]
Picroside II	HUVECs treated with homocysteine	Attenuates homocysteine-induced oxidative stress	Modulates the SIRT1/LOX-1 pathway, reduces ROS production, and rescues abnormalities of NOX, MDA, SOD, and CAT	[76]
Aucubin	HUVECs treated with ox-LDL	Improves vascular endothelial dysfunction	Reduces ROS generation and protects against eNOS uncoupling	[98]

**Table 4:** Attenuation of endothelial cell apoptosis.

Monoterpene	Experimental Model	Efficacy	Target/Pathway	Refs.
Catalpol	HUVECs treated with H_2_O_2_	Attenuates H_2_O_2_-induced apoptosis in HUVECs	Activates PI3K/AKT pathway, decreases Bax and cleaved Caspase-3, and increases Bcl-2 and p-Bad	[109]
	Human aortic endothelial cells treated with homocysteine	Protects against endothelial apoptosis induced by homocysteine	Inhibits ER stress-mediated apoptosis, enhances Bcl-2 and mitochondrial membrane potential, and reduces Bax, cleaved caspase-3, caspase-9 and cytochrome c release	[66]
Paeoniflorin	HUVECs treated with ox-LDL	Attenuates ox-LDL-induced apoptosis in HUVECs	Enhances autophagy via upregulation of SIRT1, decreases Bax, and increases Bcl-2	[64]
	HUVECs treated with TBHP	Suppresses apoptosis in HUVECs mediated by TBHP	Activates Nrf2/HO-1 signaling, decreases Bax, cleaved Caspase-3 and cytochrome c release, and increases Bcl-2	[90]
	HUVECs treated with H_2_O_2_	Protects against HUVEC apoptosis induced by H_2_O_2_	Suppresses ERK signaling and Caspase-3 activity	[89]
Geniposide	HUVECs treated with ox-LDL	Inhibits apoptosis in HUVECs subjected to ox-LDL	Modulates the miR-21/PTEN pathway, decreases Bax and Caspase-3 activity, and increases Bcl-2 and mitochondrial membrane potential	[67]
	ApoE^−/−^ mice fed with HFD and HUVECs treated with H_2_O_2_	Inhibits the growth of atherosclerosis and ameliorates H_2_O_2_-induced apoptosis in HUVECs	Modulates AMPK/mTOR/Nrf2 pathway, decreases Bax and Caspase-3, and increases Bcl-2	[68]
Picroside II	HUVECs treated with homocysteine	Protects against endothelial apoptosis induced by homocysteine	Modulates SIRT1/LOX-1 pathway, and reduces cleaved Caspase-3 as well as Caspase-3 activity	[76]
Monotropein	HUVECs treated with H_2_O_2_	Attenuates H_2_O_2_-induced apoptosis in HUVECs	Attenuates NF-κB/AP-1 signaling, decreases Bax and cleaved Caspase-3, increases Bcl-2	[77]
Albiflorin	HUVECs treated with ox-LDL	Alleviates ox-LDL-induced apoptosis in HUVECs	Blocks IRAK1/TAK1 pathway, and decreases Bax and Caspase-3	[78]
Harpagoside	bEnd.3 endothelial cells treated with Ang II	Suppresses Ang II-induced apoptosis in HUVECs	Keeps Bax/Bcl-2 balance, decreases cytochrome c release, and inactivates caspase-8, caspase-9, and caspase-3	[83]
Safranal	Bovine aortic endothelial cells treated with H_2_O_2_	Suppresses H_2_O_2_-induced endothelial apoptosis	Modulates MAPK signaling, decreases Caspase-3 and cytochrome c release, and increases Bcl-2 and survivin	[97]

**Table 5:** Reduction of macrophage-related inflammation.

Monoterpene	Experimental Model	Efficacy	Target/Pathway	Refs.
Bornyl acetate	RAW 264.7 macrophages treated with LPS	Decreases TNF-α, IL-1β, and IL-6	Suppresses p38 MAPK/JNK/ERK signaling and IκBα/NF-κB signaling	[128]
Eucalyptol	Murine peritoneal macrophages, BMDMs, or alveolar macrophages treated with LPS or LPS+ATP	Decreases TNF-α, IL-1α, IL-1β, IL-6, COX-2, iNOS, and NO and increases IL-10	Suppresses NF-κB, JNK, p38 MAPK, STAT3, and NLRP3 inflammasome activation	[129,130,131]
α-Pinene	Murine peritoneal macrophages treated with LPS	Decreases IL-6, TNF-α, COX-2, iNOS, PGE_2_, and NO	Suppresses JNK, ERK, and IKK/NF-κB signaling	[132]
Geraniol	RAW 264.7 macrophages treated with LPS	Decreases COX-2, iNOS, PGE_2_, and NO	Suppresses IκBα/NF-κB signaling	[133]
Linalool	RAW 264.7 macrophages treated with LPS	Decreases TNF-α and IL-6	Suppresses p38 MAPK/JNK/ERK and IκBα/NF-κB signaling	[134]
Citral	Murine alveolar macrophages or J774A.1 macrophages treated with LPS or LPS+ATP	Decreases TNF-α, IL-1β, and IL-6	Regulates PPAR-γ/NF-κB signaling and inhibits NLRP3 inflammasome activation	[119,135]
Citronellol	RAW 264.7 macrophages treated with LPS	Decreases COX-2, iNOS, PGE_2_, and NO	Suppresses IκBα/NF-κB signaling	[133]
Carvacrol	THP-1 cells, J774A.1 cells, or macrophage-like U937 cells treated with LPS or LPS+ATP	Decreases TNF-α, IL-1β, IL-18, and COX-2	Suppresses NF-κB, JNK, ERK, STAT-3, AP-1, NFATs, and NLRP3 inflammasome and activates PPAR-γ	[120,136,137]
Thymol	RAW 264.7 macrophages treated with LPS	Decreases TNF-α, IL-6, COX-2, and NO	Suppresses NF-κB, MAPK, STAT-3, AP-1, and NFATs	[137,138]
Perillaldehyde	RAW 264.7 macrophages treated with LPS	Decreases TNF-α, IL-1β, and IL-6	Suppresses JNK signaling	[139]
Hinokitiol	RAW 264.7 macrophages treated with LPS	Decreases TNF-α	Suppresses the phosphorylation of PDK1, AKT/PKB, and ERK and consequently reduces NF-κB activation	[140]
Carvone	RAW 264.7 macrophages treated with LPS	Anti-inflammatory effect	Suppresses JNK signaling and promotes SIRT1-mediated NF-κB-p65 deacetylation	[116]
p-Cymene	RAW 264.7 macrophages treated with LPS	Decreases TNF-α, IL-1β, and IL-6	Suppresses p38 MAPK/JNK/ERK signaling and IκBα/NF-κB signaling	[141]
Thymoquinone	RAW 264.7 macrophages treated with LPS	Decreases iNOS, COX-2, TNF-α, IL-1β, and IL-6	Suppresses IRAK1-linked AP-1/NF-κB pathways	[142]
Gamma-Terpinene	Murine peritoneal macrophages treated with LPS	Decreases IL-1β and IL-6 and enhances IL-10	Promotes the PGE_2_/IL-10 axis	[143]
Safranal	RAW 264.7 macrophages or J774A.1 cells treated with LPS or LPS+ATP	Decreases TNF-α, IL-1β, IL-6, COX-2, iNOS, and NO	Suppresses MAPK/AP-1 and IKK/NF-κB signaling and inhibits NLRP3 inflammasome activation	[144,145]
Geniposide	ApoE^−/−^ mice fed with HFD, RAW 264.7 macrophages or primary mouse macrophages treated with LPS or LPS+ATP	Attenuates atherosclerosis and decreases TNF-α, IL-1β, and IL-6	Modulates miR-101/ MKP-1/p38, TLR4-mediated NF-κB and MAPK signaling, and AMPK/SIRT1/NLRP3 inflammasome activation	[113,114,115]
Genipin	RAW 264.7 macrophages treated with LPS	Decreases NO, iNOS and COX-2	Suppresses IκBβ/NF-κB and promotes PI3K/JNK/Nrf2/HO-1 signaling	[121,146]
Catalpol	Murine alveolar macrophages or THP-1 cells treated with LPS	Decreases TNF-α, IL-1β, IL-6, and IL-4 and increases IL-10	Suppresses NLRP3 inflammasome activation and TLR4-mediated NF-κB and MAPK signaling	[147,148]
Swertiamarin	RAW 264.7 macrophages treated with LPS	Decreases TNF-α, IL-1β, IL-6, IL-8, iNOS, and COX-2 and increases IL-10 and IL-4	Suppresses IκBα/NF-κB and JAK2/STAT3 signaling and targets the AKT-PH domain to reduce the phosphorylation of AKT	[149,150]
Paeoniflorin	RAW 264.7 macrophages or THP-1 cells treated with AGEs or LPS	Decreases TNF-α, IL-1β, IL-6, IL-33, MCP-1, and iNOS	Suppresses miR-124, TLR2/4, NF-κB and p38 MAPK with the regulation of Ca^2+^ mobilization and modulates the SOCS3-ASK1-p38 pathway	[127,151,152,153]
MBPF	RAW 264.7 macrophages treated with LPS	Decreases TNF-α, IL-6, iNOS, and NO	Suppresses NF-κB, MAPK and PI3K/AKT	[154]
Cornuside	RAW 264.7 macrophages treated with LPS	Decreases TNF-α, IL-1β, IL-6, iNOS, COX-2, NO, and PGE_2_	Suppresses IκBα/NF-κB and MAPK signaling	[155]
Loganin	Mice fed with dextran sulfate sodium, RAW 264.7 macrophages, or BMDMs treated with LPS or LPS+MSU	Decreases TNF-α, IL-1β, IL-6, MCP-1, CXCL10, iNOS, COX-2, NO, and PGE_2_	Suppresses SIRT1/NF-κB and mitochondrial dysfunction-mediated NLRP3 inflammasome activation and induces Nrf2/HO-1 signaling	[117,122,156]
Oleuropein	J774A.1 macrophages treated with LPS	Decreases TNF-α, IL-6, iNOS, COX-2, and NO	Modulates CD14/TLR4-MyD88-NF-κB/MAPK pathways	[157]
Oleacein	THP-1 cells treated with LPS	Decreases TNF-α, IL-1β, IL-6, NO, and PGE_2_ and increases IL-10	Suppresses TLR4/MyD88/NF-κB Pathway	[158]
Oleocanthal	Mouse peritoneal Macrophages treated with LPS	Decreases TNF-α, IL-1β, IL-6, IL-17, IL-18, INF-γ, iNOS, COX-2, NO, and PGE_2_	Activates Nrf2/HO-1 and inhibits MAPK and NLRP3 inflammasome activation	[123]
Picroside II	THP-1 cells treated with LPS+ATP	Decreases IL-1β	Suppresses NF-κB-mediated NLRP3 inflammasome activation	[159]
Gentiopicroside	RAW 264.7 macrophages or primary mouse macrophages treated with LPS+INF-γ or LPS+MSU	Decreases TNF-α, IL-1β, IL-6, IL-18, CCL-5, CXCL10, and iNOS	Suppresses IKKα/β/NF-κB signaling and NLPR3 inflammasome activation	[160,161]
Aucubin	RAW 264.7 macrophages and THP1 cells treated with LPS	Decreases TNF-α, IL-1β, iNOS, and COX-2	Induces the AMPK/Nrf2 pathway	[124]
Harpagoside	RAW 264.7 macrophages treated with LPS	Decreases iNOS and COX-2	Suppresses IκBα/NF-κB signaling	[162]
Scropolioside B	THP-1 cells treated with LPS	Decreases TNF-α, IL-1β, and IL-32	Suppresses NLRP3 inflammasome activation	[147]
Catalposide	RAW 264.7 macrophages treated with LPS	Decreases TNF-α, IL-1β, and IL-6	Suppresses the binding of LPS to CD14 on the surface of cells thereby inhibiting NF-kB signaling	[163]
Monotropein	RAW 264.7 macrophages treated with LPS	Decreases TNF-α, IL-1β, iNOS, COX-2, NO, and PGE_2_	Suppresses IKKβ/NF-κB and MAPK signaling	[164]
Asperulosidic Acid	RAW 264.7 macrophages treated with LPS	Decreases TNF-α, IL-6, iNOS, COX-2, NO, and PGE_2_	Suppresses JNK, ERK, and IκBα/NF-κB signaling	[165]
Asperuloside	RAW 264.7 macrophages treated with LPS	Decreases TNF-α, IL-6, iNOS, COX-2, NO, and PGE_2_	Induces Nrf2/HO-1 and suppresses MAPK and IκBα/NF-κB signaling	[125,165]
Sweroside	BMDMs or RAW 264.7 macrophages treated with LPS	Decreases TNF-α, IL-1β, IL-6, COX-2, iNOS, PGE_2_, and NO and increases IL-10	Increases SIRT1 signaling and suppresses NLRP3 inflammasome activation	[118,166]
Nuezhenide	RAW 264.7 macrophages treated with LPS	Decreases TNF-α, IL-6, iNOS, COX-2, and NO	Suppresses IKKα/β/NF-κB signaling	[167]
Morroniside	RAW 264.7 macrophages treated with LPS	Decreases TNF-α, IL-1β, iNOS, COX-2, NO, and PGE_2_	Modulates TLR4/NF-κB and Nrf2/HO-1 signaling	[126]
Scandoside	RAW 264.7 macrophages treated with LPS	Decreases TNF-α, IL-6, iNOS, COX-2, NO, and PGE_2_	Suppresses IκBα/NF-κB and MAPK signaling	[168]
Mussaenoside	RAW 264.7 macrophages treated with LPS	Decreases TNF-α, IL-1β, iNOS, COX-2, NO, and PGE_2_	Suppresses NF-κB signaling	[169]

**Table 6:** Modulation of foam cell formation, autophagy and M2 macrophage polarization.

Monoterpene	Experimental Model	Efficacy	Target/Pathway	Refs.
Isoborneol	RAW 264.7 macrophages treated with ox-LDL	Reduces the absorption of ox-LDL and the accumulation of intracellular lipids	Modulation of cell migration and polarity-related pathways may be involved	[185]
	RAW 264.7 macrophages treated with LPS+INF-γ or IL-4+IL-13	Promotes M2 macrophage polarization as shown by elevated expression of CD206, Arg-1 and IL-10	Activates the JAK2-STAT3 signaling pathway	[186]
Eucalyptol	THP-1 or RAW 264.7 macrophages treated with ox-LDL	Suppresses foam cell formation and promotes cholesterol efflux	Upregulates the expression of LXRs and their target genes ABCA1 and ABCG1	[182,183]
Geniposide	ApoE^−/−^ mice fed with HFD, RAW 264.7 cells treated with LPA	Inhibits atherosclerosis and attenuates foam cell formation by regulating both lipid uptake and efflux	Suppresses p38 MAPK and AKT signaling pathways	[184]
	BMDMs treated with ox-LDL	Inhibits foam cell formation and inflammatory response	Suppresses CD36 expression and NF-κB and MAPK signaling pathways	[179]
	New Zealand rabbits fed with HFD	Inhibits atherosclerosis, suppresses M1 macrophage polarization, and promotes M2 polarization	Suppresses the FOS/ MAPK signaling pathway	[187]
	ApoE^−/−^ mice fed with HFD, RAW 264.7 cells treated with ox-LDL	Inhibits the progression of atherosclerosis and reinforces macrophage autophagy	Suppresses the TREM2/mTOR axis	[188]
Genipin	BMDMs (M0, M1, M2-type)	M2 polarization induction and maintenance, along with suppressed pro-inflammatory M1/iNOS response	Activates the pSTAT6/PPARγ pathway	[189]
Catalpol	Postmenopausal atherosclerosis mouse model, J774A-1 macrophages treated with LPS+INF-γ	Prevents postmenopausal atherosclerosis, suppresses M1 macrophage polarization and promotes M2 polarization	Increases the expression of ERα	[190]
Paeoniflorin	Mouse BMDMs treated with LPS or IL-4	Suppresses M1 macrophage polarization and promotes M2 polarization	Decreases NF-κB and increases STAT6 signaling	[191]
	Mouse peritoneal macrophages treated with ox-LDL	Attenuates ox-LDL-induced foam cell formation	Suppression of NF-κB, ERK and p38 MAPK may be involved	[192]
Albiflorin	THP-1 cells treated with ox-LDL	Blocks foam cell formation	Modulates the LOX-1/NF-κB signaling pathway	[180]
Loganin	RAW 264.7 macrophages or peritoneal macrophages treated with LPS	Suppresses M1 macrophage polarization and promotes M2 polarization	Suppresses ERK and NF-κB signaling	[193]
Oleacein	Human monocyte-derived macrophages treated with ox-LDL	Decreases foam cell formation, reduces apoptosis, and shifts the polarization towards M2 macrophage phenotype	Suppresses the expression of CD36, SRA1 and LOX-1 and activates JAK/STAT3 pathway	[177]
	Human monocyte-derived macrophages treated with hemoglobin/haptoglobin complexes	Enhances M2 macrophage phenotype	Increases the expression of CD163 and IL-10 receptors as well as HO-1	[194]

**Table 7:** Modulation of VSMC proliferation and migration and inhibition of dendritic cell maturation.

Monoterpene	Experimental Model	Efficacy	Target/Pathway	Refs.
Linalool	Rat aortic VSMCs (A7r5) treated with Ang II	Inhibits Ang II-induced VSMC proliferation and migration	Suppresses CHRM3-mediated p38 MAPK/JNK/ERK signaling	[208]
Carvacrol	Rat aortic VSMCs treated with PDGF-BB	Inhibits VSMC proliferation and migration and attenuates atherosclerotic neointima formation	Suppresses ROS-mediated p38 MAPK/ERK signaling	[209]
	Mouse splenic dendritic cells	Inhibits dendritic cell maturation and adaptive immunity	Suppresses CD40	[217]
Hinokitiol	Rat aortic VSMCs treated with PDGF-BB	Inhibits the PDGF-BB-stimulated proliferation of VSMCs	Suppresses JNK and PLC-γ1 and induces p27^kip1^ expression	[215]
Thymoquinone	Rat aortic VSMCs treated with Ang II or PDGF-BB	Inhibits VSMC proliferation, migration, and neointimal formation and promotes VSMC apoptosis	Promotes AMPK/PPARγ/PGC-1α and inhibits p38 MAPK and MMP-2	[211,212]
	BMDCs treated with LPS	Inhibits maturation, cytokine release and survival of dendritic cells	Suppresses CD11c, CD86, MHCII, CD54, and CD40 and reduces PI3K/AKT and ERK signaling	[218]
Genipin	Rat aortic VSMCs treated with TNF-α	Inhibits TNF-α-induced VSMC proliferation and migration	Suppresses ERK and AKT signaling via upregulating HO-1 expression	[214]
Paeoniflorin	Rat aortic VSMCs treated with ox-LDL or PDGF-BB	Inhibits VSMCs proliferation, migration and inflammation	Activates HO-1 and then inhibits ROS-mediated p38 MAPK, ERK and NF-κB pathways	[192,213]
	BMDCs or monocyte-derived dendritic cells treated with LPS	Inhibits dendritic cell function, impairs Th17 cell differentiation, and generates regulatory dendritic cells	Reduces IKK/NF-κB and JNK-mediated IL-6 and costimulatory molecule expression and induces TGF-β/IDO signaling	[219,220]
Oleuropein	Bovine vascular SMCs	Inhibits SMC proliferation with a cell cycle block between the G1 and the S phases	Suppresses ERK signaling	[221]
Plumericin	Rat aortic VSMCs stimulated with serum	Arrested VSMCs in the G1/G0-phase of the cell cycle	Suppresses STAT3 signaling via S-glutathionylation	[216]
Thymol	Mouse splenic dendritic cells	Inhibits dendritic cell maturation and adaptive immunity	Suppresses CD86	[217]
Geniposide	ApoE^−/−^ mice fed with HFD	Attenuates atherosclerosis and inhibits dendritic cell maturation in bone marrow and infiltration into lesions	Suppresses CD11c, CD80, CD86, and CD83 in bone marrow or in atherosclerotic lesions	[222]

## Data Availability

Not applicable.
